# Chemical Composition and Larvicidal Activity of Essential Oils Extracted from Brazilian Legal Amazon Plants against *Aedes aegypti* L. (Diptera: Culicidae)

**DOI:** 10.1155/2015/490765

**Published:** 2015-04-09

**Authors:** Clarice Noleto Dias, Luciana Patrícia Lima Alves, Klinger Antonio da Franca Rodrigues, Maria Cristiane Aranha Brito, Carliane dos Santos Rosa, Flavia Maria Mendonça do Amaral, Odair dos Santos Monteiro, Eloisa Helena de Aguiar Andrade, José Guilherme Soares Maia, Denise Fernandes Coutinho Moraes

**Affiliations:** ^1^Programa de Pós-Graduação em Saúde e Ambiente, Universidade Federal do Maranhão, 65085-580 São Luís, MA, Brazil; ^2^Departamento de Farmácia, Universidade Federal do Maranhão, 65085-580 São Luís, MA, Brazil; ^3^Programa de Pós-Graduação em Química, Universidade Federal do Maranhão, 65085-580 São Luís, MA, Brazil; ^4^Programa de Pós-Graduação em Química, Universidade Federal do Pará, 66075-900 Belém, PA, Brazil; ^5^Programa de Pós-Graduação em Recursos Naturais da Amazônia, Universidade Federal do Oeste do Pará, 68035-110 Santarém, PA, Brazil

## Abstract

The mosquito *Aedes aegypti* L. (Diptera: Culicidae) is the major vector of dengue and chikungunya fever. The lack of effective therapies and vaccines for these diseases highlights the need for alternative strategies to control the spread of virus. Therefore, this study investigated the larvicidal potential of essential oils from common plant species obtained from the Chapada das Mesas National Park, Brazil, against third instar *A. aegypti* larvae. The chemical composition of these oils was determined by gas chromatography coupled to mass spectrometry. The essential oils of *Eugenia piauhiensis* Vellaff., *Myrcia erythroxylon* O. Berg, *Psidium myrsinites* DC., and *Siparuna camporum* (Tul.) A. DC. were observed to be mainly composed of sesquiterpene hydrocarbons. The essential oil of *Lippia gracilis* Schauer was composed of oxygenated monoterpenes. Four of the five tested oils were effective against the *A. aegypti* larvae, with the lethal concentration (LC_50_) ranging from 230 to 292 mg/L after 24 h of exposure. Overall, this work demonstrated the possibility of developing larvicidal products against *A. aegypti* by using essential oils from the flora of the Brazilian Legal Amazon. This in turn demonstrates the potential of using natural resources for the control of disease vectors.

## 1. Introduction

The Brazilian Legal Amazon encompasses approximately 5 million square kilometers. This area is going through a serious deforestation process and the state of Maranhão has one of the highest rates [1, 2]. An important step towards protecting its biodiversity was the creation of the Chapada das Mesas National Park, located in the central-southern region of the state [3].

Another important way towards the preservation of biodiversity is the search for plant products that could be used in order to provide a sustainable development of the Amazon [4]. Although several research groups have been devoted to the study of phytotherapeutic agents, phytocosmetics, and medicinal plants in this region, Frickmann and Vasconcellos [5] have reported that the research conducted has not contributed to the development of many innovative products so far.

The use of essential oils as insecticides is a highly promising initiative to develop and preserve the region [6–8]. They are known to be complex mixtures of secondary metabolites that can be obtained at low costs using renewable technology, often displaying higher activities than the individual isolated compounds [9].

Some acute virus diseases, such as dengue and chikungunya fever, are transmitted through the bite of female* Aedes (Stegomyia) aegypti* L. (1762) (Diptera: Culicidae) mosquitoes [10, 11]. Dengue fever has been classified as the most important disease caused by arbovirus in the world [12]. Chikungunya fever has seen unprecedented global expansion during 2004, with epidemics recorded in Africa, Asia, and the islands of the Indian Ocean and in areas with temperate climate, such as Europe [13], and has been overspread quickly in Brazil, where there were 1,750 reported cases of this illness in 2014 [14]. Despite extensive research, these diseases currently have no treatment or any officially approved vaccine [15].

Preventing the spread of these viruses is, consequently, dependent on the eradication of their main vector, the arthropod* A. aegypti* [16]. Chemical control, through the use of insecticides, is the approach most commonly employed by public health initiatives [17]. Focal treatment, which involves the eradication of immature forms of* A. aegypti* by using larvicides (such as the organophosphate temefos) in the specific places where the larvae can be found, is a very efficient approach [18, 19]. However, the regular and repeated use of these insecticides has resulted in the development of resistant strains [20].

Although some previous studies have been performed with the aim to evaluate the larvicidal activity of essential oils, in general, these studies are not conducted with the ultimate goal of producing plant-based larvicides [9]. This study is focused on the sustainable use of Amazonian aromatic plants and the development of new products to combat the spread of the dengue and chikungunya virus. The main aim of this study was to test the larvicidal activity against* A. aegypti* of essential oils from five common plant species found in the Brazilian Legal Amazon, plants that have been poorly studied so far, as well as to determine the chemical composition of these oils, in order to establish the quality parameters of the bioproducts.

## 2. Materials and Methods 

### 2.1. Plant Material

The collection of aromatic plants was based on conformance to standard sensorial criteria and the observation of morphological traits; the inspections were performed by collaborating botanists present during sample collection to determine if a particular sample belonged to one of the families of aromatic plants. The inclusion criteria favored the inclusion of poorly researched plant species.

Leaves were collected from adult specimens of the selected plants in the early morning. The plants were endemic to several regions within the Chapada das Mesas National Park, Maranhão, Brazil, as well as several buffer zones of the Park. This area was a constituent of the Unit of Environmental Conservation and included Amazonian plants and plants found in the Cerrado region of the state of Maranhão. The permission for plants collection (process number: 28007-2) was provided by the System of Authorization and Information in Biodiversity of the Chico Mendes Institute of Conservation and Biodiversity (Instituto Chico Mendes de Conservação da Biodiversidade (ICMBio)), Ministry of Environment, Brazil.

The species identification was performed at the Herbarium João Murça Pires of the Emílio Goeldi Pará Museum, Belém, Pará, Brazil. The complete list of collected species and the geographical coordinates, dates, and records of collections are provided in Table 1.

### 2.2. Plant Processing and Extraction of the Essential Oils

The leaves of collected plants were air-dried, ground, and submitted to hydrodistillation, using a Clevenger-type apparatus (100 g each, 3 h). The oils were dried over anhydrous sodium sulfate. The percentage content of oil was calculated based on plant dry weight.

### 2.3. Essential Oils-Composition Analyses

The analyses of the oils were carried out on a THERMO DSQ II GC-MS instrument (Thermo Fisher Scientific, Austin, TX, USA), under the same conditions previously described [23].

Individual components were identified by comparison of both mass spectrum and GC retention data with authentic compounds, which were previously analyzed and stored in a private library [21], as well as with the aid of commercial libraries containing retention indices and mass spectra of volatile compounds commonly found in essential oils [22, 24].

### 2.4. Larvicidal Activity Assay

*A. aegypti* eggs were collected using ovitraps [25] that were distributed in neighborhoods with high incidence of dengue in São Luís, Maranhão, Brazil. The pallets containing the eggs were submerged in dechlorinated tap water in order to allow hatching of the eggs; ground Purina Cat Chow (0.3 g) was added to the water to improve the conditions for larval development. Larvae were incubated in order to achieve the third instar stage, the optimal bioassay phase. Larvae were grown at controlled temperatures (25 ± 2°C) with a 12 h photoperiod. The larvae were identified using Consoli and Oliveira keys [26].

The larvicidal activity bioassay was performed according to the method proposed by the World Health Organization, with slight modifications [27]. The stock solutions of each essential oil sample were prepared in mineral water and 0.01% dimethyl sulfoxide (DMSO). Aliquots of the stock solutions were used to prepare solutions of six different concentrations (20 mL; 50, 100, 200, 300, 400, and 1000 mg/L). Ten larvae were added to each solution. The mortality was scored 24 h after the start of the experiment. A 0.01% DMSO solution and a 1 mg/L temefos solution were used as the negative and positive control, respectively. Each assay was performed in triplicate (repeated three times on different days).

### 2.5. Statistical Analyses

The half-maximal lethal concentration (LC_50_) value was calculated using probit regression model (SPSS program, version 13.0) and assuming a confidence level of 95% (*P* < 0.05).

## 3. Results and Discussion

### 3.1. Analysis of the Essential Oil Composition

The essential oils were extracted from the leaves of* Eugenia piauhiensis *Vellaff.,* Myrcia erythroxylon* O. Berg,* Psidium myrsinites *DC.,* Siparuna camporum *(Tul.) A. DC., and* Lippia gracilis* Schauer at yields of 0.43, 0.53, 0.39, 1.40, and 7.99% (v/w), respectively. The density of all extracted oils was less than that of water, while the oil color was light yellow. Fifty-six components, representing 91.94 to 99.64% of the oils, were identified (Table 2).

Sesquiterpene hydrocarbons were the major identified components in the oils of* E. piauhiensis*,* M. erythroxylon*,* P. myrsinites,* and* S. camporum*, whereas 1,8-cineole and *α*-terpineol oxygenated monoterpenes were the major components observed in* L. gracilis.* The major components of* E. piauhiensis*,* M. erythroxylon*,* P. myrsinites*, and* S. camporum* were *γ*-elemene and (*E*)-*β*-caryophyllene, germacrene D and bicyclogermacrene, (*E*)-*β*-caryophyllene and *α*-humulene, and *γ*-patchoulene and *α*-phellandrene, respectively.

The composition and yield of the essential oils of each plant species may vary according to the genetic, environmental, and physiopathological traits influencing the plant metabolism. The total quantity, as well as the relative proportions of secondary metabolites in plants, can be regulated by biotic and abiotic factors, such as the season, area, and time of collection of the samples [28–31]. This demonstrates the relevance of chemical and biochemical studies using plants collected from different regions, during different times of the year.

Maia et al. [32] revealed that* P. myrsinites* leaves, collected from Anapurus, in the Maranhão region of the Amazon, were mainly composed of (*E*)-*β*-caryophyllene (28.7%) and *α*-humulene (19.4%), which is in agreement with the results presented in this study. The similarities between the physical profile of this species and the species identified in this study may be because the plants (in both studies) were collected from regions with identical climates.

In contrast, the essential oil of* L. gracilis* was observed to be mainly composed of 1,8-cineole in this study, which does not correspond to the results of previous reports; the essential oil of the leaves of this species collected from São Felix de Balsas, Maranhão, was determined to contain over 70% of thymol [33]. Other studies have reported carvacrol to be the major component of this essential oil; however, the oil was extracted from samples collected in other states of the Brazilian northeastern region, such as Ceará and Pernambuco [34, 35]. Mendes et al. [36] identified thymol (24.1%),* p*-cymene (15.9%), and 1,8-cineole (4.8%) to be the major chemical components of the essential oil of* L. gracilis* obtained from São Cristovão, Sergipe, in Brazil.

These results highlight the need for testing the composition of essential oils using analytical techniques. The biological properties of the plants are known to depend on their composition, independent of their products (essential oils, fixed oils, latex, resins, or extracts). Among the numerous plant-derived products, essential oils are considered to have the most variable composition because of external (soil, climate, and altitude) and internal (age, portion of the plant) factors [37, 38]. As such, the study of the chemical composition of essential oils from aromatic plants is fundamental to the scientific understanding of plant-derived products and may lay the foundation for the development of human-oriented assets.

### 3.2. Larvicidal Activity

The essential oil obtained from* M. erythroxylon* was considered to be inactive since it did not kill mosquito larvae, even at the highest concentration studied (1000 mg/L); the remaining essential oils were considered to be effective (LC_50_ between 230 and 292 mg/L, after 24 h of exposure) (Table 3).

The highest concentration studied for the effective essential oils was 400 mg/L. The larvae subjected to this concentration were agitated during the first 30 min of exposure; following this, the larvae showed slowed and abnormal movements, including tremors and convulsions. The larvae were afflicted with paralysis and floated to the bottom of the containers prior to death. The mortality rate was observed to be directly proportional to the tested concentrations.

*Aedes aegypti* L. is an important vector of dengue and chikungunya fever, diseases that still cause high morbidity and mortality in several countries of the world [39]. The lack of effective vaccines and specific therapies for these diseases has prompted the search for new approaches to control mosquito population, notably because of the increase in mosquito resistance to conventional pesticides [40, 41]. The observation that essential oils could be used as effective natural insecticides [42, 43] has inspired a series of studies, which have confirmed the biological larvicidal potential of essential oils against* A. aegypti *larvae [9, 44].

This study tested the larvicidal potential of selected essential oils against* A. aegypti*. This study utilized the procedures proposed by the WHO [27], which establishes the protocols to be used for the study of efficacy; however, the recommendations did not define the larval stage, or the time of exposure for the assays. Therefore, these studies cannot be standardized, which has complicated the comparison of results between different interspecies and intraspecies studies.

Another important factor that is not usually discussed in scientific reports is the origin of the larvae used (field-collected or laboratory-reared). In general, the field-collected larvae are more resistant to pesticides compared to those reared under laboratory conditions, as the former are better adapted to adjust to environmental variations and express higher genetic variability [45, 46]. The same compound ((*R*)-(-)-carvone) showed different larvicidal potential against larvae reared in the laboratory for more than 10 years and those collected in fields (known to have larval strains resistant to temefos) [47, 48].

Since the WHO has not established a standard criterion for determining the larvicidal activity of natural products, several authors have developed individual criteria to characterize the potency of mosquito larvicides developed from natural products [44, 49, 50]. Komalamisra et al. [51] considered products showing LC_50_ ≤ 50 mg/L to be active, 50 mg/L < LC_50_ ≤ 100 mg/L to be moderately active, 100 mg/L < LC_50_ ≤ 750 mg/L to be effective, and LC_50_ > 750 mg/L to be inactive. Ravi Kiran et al. [52] considered compounds with LC_50_ < 100 mg/L to exhibit a significant larvicidal effect. It should be stressed that these criteria must be directly correlated with the time of exposure and the origin of larvae, which are variables that can alter the LC_50_ values.

The results obtained in this study showed that four of the five plant species had promising effects, according to the criterion established by Komalamisra et al. [51], exhibiting LC_50_ between 230 and 292 mg/L after 24 h of exposure. These values were obtained for the essential oils extracted from* E. piauhiensis*,* P. myrsinites*,* S. camporum*, and* L. gracilis*.

The oil extracted from the leaves of* L. gracilis* has been previously researched. The studies conducted by Santiago et al. [53] and Silva et al. [54] showed lower LC_50_ values than the ones observed in this work. However, the experimental conditions were considerably different, especially compared to those utilized by Silva et al. [54], who used laboratory-reared larvae. In addition, it must be emphasized that the chemical composition of these oils may vary; the previous study indicated that the oxygenated monoterpene, carvacrol, was the major component found in this species, while this study identified 1,8-cineole to be the major component.

The essential oils extracted from* E. piauhiensis *and* S. camporum* showed the most promising larvicidal potential, with lower LC_50_ values. The essential oils of the* Myrtaceae* species were, in the past, believed to be active against* A. aegypti *larvae [7]. Essential oils from other species belonging to the genera* Eugenia *L. and* Psidium* L. are also considered to be active against* A. aegypti *larvae. These include* Eugenia melanadenia* Krug & Urb.,* Psidium guajava *L., and* Psidium rotundatum *Griseb. [55, 56].

The* M. erythroxylon* extract was the only essential oil considered inactive as a larvicide in this study. In contrast, the essential oil extracted from leaves of another species of the same genus,* M. ovata *Cambess., showed larvicidal activity [56]. This is one example highlighting that the results of such studies should not be correlated with the chemical-taxonomic classification. This is especially true in the case of essential oils; the chemical-taxonomic approach is not always an adequate tool for the selection of plants to be researched.

## 4. Conclusions

The use of plant-derived products, such as essential oils, in the production of natural larvicidal insecticides, could be a promising tool to help reduce the spread of dengue and chikungunya fever. This is because these products are the natural sources of substances displaying insecticidal activity against mosquito (affecting the different stages of mosquito development). In addition, these products are biodegradable and express low toxicity towards nontarget organisms.

However, it is important to standardize the procedures used for the determination of larvicidal activity. To this effect, the WHO must establish specific procedures for the control or elimination of* A. aegypti* larvae.

The* A. aegypti* larvae were observed to be sensitive to four of the five essential oils tested in this study. In light of these results, we stress the importance of describing the chemical composition and biological properties of essential oils. This would help in the development of products with larvicidal activities from Amazonian plants. It should also be stressed that any product developed should be accompanied by a rational and sustainable production system, in order to protect the Amazonian biodiversity.

## Figures and Tables

**Table 1:** Plant species collected from the Chapada das Mesas National Park, central-southern region of the state of Maranhão, Brazil.

Family and plant species	Geographical coordinates	Dates	Records of collections
Myrtaceae			
*Eugenia piauhiensis* Vellaff.	07°19′14.8′′ S; 47°19′14.8′′ W	02/28/2012	L-3146
*Myrcia erythroxylon* O. Berg	07°09′32.7′′ S; 47°24′05.1′′ W	07/20/2011	L-2907
*Psidium myrsinites* DC.	07°06′3.65′′ S; 47°20′35.3′′ W	02/28/2012	L-3152
Siparunaceae			
*Siparuna camporum* (Tul.) A. DC.	07°03′04.6′′ S; 47°27′09.1′′ W	03/01/2012	L-3174
Verbenaceae			
*Lippia gracilis* Schauer	07°06′3.65′′ S; 47°20′35.3′′ W	02/28/2012	L-3151

**Table 2:** Main constituents of the essential oils (of plants obtained from the Brazilian Legal Amazon) tested against *Aedes aegypti* L. (Diptera: Culicidae) larvae.

Compounds^a^/classes	RI^b^	Plant species/relative content^c^ (%)				
		Epi	Me	Pm	Sc	Lg
*α*-Pinene	931	3.54	—	—	—	—
Sabinene	977	—	—	—	—	2.72
*β*-Pinene	977	7.08	—	—	—	6.22
Myrcene	989	—	—	1.32	—	2.2
*α*-Phellandrene	1003	—	—	—	12.8	—
*α*-Terpinene	1017	—	—	—	—	0.92
*p*-Cymene	1024	—	—	—	1.5	1.35
Limonene	1029	1.27	—	—	4.9	—
1,8-Cineole	1034	—	—	0.66	—	56.16
(*E*)-*β*-Ocimene	1046	5.52	—	—	—	—
*γ*-Terpinene	1059	—	—	—	—	3.55
*α*-Terpinolene	1085	—	—	—	0.41	—
Linalool	1100	—	—	0.78	—	—
*trans*-Pinocarveol	1140	—	—	—	—	3.42
*δ*-Terpineol	1167	—	—	—	—	1.02
Terpinen-4-ol	1180	—	—	—	—	3.83
*α*-Terpineol	1186	0.43	—	—	—	12.09
Geraniol	1256	—	—	—	0.49	—
(*E*)-Linalool oxide acetate	1286	—	—	—	0.46	—
*δ*-Elemene	1335	3.49	—	—	4.9	—
*α*-Copaene	1378	1.5	1.85	—	0.38	—
*β*-Bourbonene	1386	0.57	—	—	—	—
*β*-Elemene	1390	4.79	2.41	—	3.29	—
*α*-Gurjunene	1412	—	0.52	0.43	—	—
(*E*)-*β*-Caryophyllene	1419	16.46	10.55	26.05	3.17	1.12
*β*-Gurjunene	1432	—	4.71	—	3.36	—
*γ*-Elemene	1434	17.48	5.38	—	—	—
*α*-Guaiene	1438	—	—	—	1.94	—
Aromadendrene	1440	2.29	1.06	1.4	—	—
Guaiadiene-6,9	1444	—	—	—	9.23	—
*trans-*Muurola-3,5-diene	1450	—	—	—	1.47	—
*α*-Humulene	1455	2.18	—	23.92	0.69	1.49
*allo*-Aromadendrene	1462	—	2.64	—	—	—
Bicyclogermacrene	1470	8.11	13.26	—	5.11	—
*γ*-Muurolene	1480	0.91	—	0.63	—	—
Germacrene D	1482	5.64	26.79	—	—	—
*γ*-Patchoulene	1484	—	—	—	28.63	—
*β*-Selinene	1487	1.23	—	1.95	1.6	—
*α*-Selinene	1496	—	—	1.25	—	—
Viridiflorene	1497	1.42	—	—	—	—
*α*-Muurolene	1501	—	—	—	3.76	—
*δ*-Cadinene	1523	2.95	9.63	—	0.72	—
Zonarene	1526	—	—	3.53	—	—
*α*-Cadinene	1539	—	1.22	—	—	—
Selina-3,7(11)-diene	1542	—	—	2.05	0.57	—
Germacrene B	1558	1.99	—	—	—	—
Spathulenol	1578	2.5	3.25	—	1.33	—
Caryophyllene oxide	1581	—	—	10.09	—	—
Globulol	1585	—	4.16	—	—	—
Viridiflorol	1590	2.33	—	2.19	5.02	—
Humulene epoxide II	1601	—	—	6.37	—	—
*epi*-Cubenol	1626	—	—	—	2.14	—
Caryophylledienol II	1631	—	—	5.66	—	—
*epi*-*α*-Cadinol	1639	—	—	2.73	—	—
*τ*-Muurolol	1640	0.81	2.26	—	0.36	—
*α*-Cadinol	1654	1.05	2.25	3.81	1.09	—

Monoterpene hydrocarbons		17.41	—	1.32	19.61	16.96
Oxygenated monoterpenes		0.43	—	1.44	0.95	81.07
Sesquiterpene hydrocarbons		71.01	80.02	61.21	68.87	2.61
Oxygenated sesquiterpenes		6.69	11.92	30.85	9.94	—
Total		**95.54**	**91.94**	**94.82**	**99.37**	**99.64**

^a^Compounds listed in order of elution on the DB-5ms column.

^b^Retention indices (RIs) experimentally determined against n-alkanes by using the DB-5ms column.

^c^Content expressed as percentages obtained by integration of the GC peak area.

RI: retention index; Epi: *Eugenia piauhiensis* Vellaff.; Me: *Myrcia erythroxylon* O. Berg; Pm: *Psidium myrsinites* DC.; Sc: *Siparuna camporum* (Tul.) A. DC.; Lg: *Lippia gracilis *Schauer. Conditions of analysis: gas chromatograph associated with mass spectrometer THERMO DSQ II; chemical constituents identified by comparison of the mass spectra obtained with published spectra [21, 22].

**Table 3:** Lethal concentrations for 50% of the test subjects (*Aedes aegypti* L.; *n* = 10) (LC_50_) of the essential oils obtained from plant species endemic to the Brazilian Legal Amazon after 24 h of exposure.

Plant species	LC_50_^a^ (mg/L)
*Eugenia piauhiensis* Vellaff.	230 (194–306)^b^
*Myrcia erythroxylon* O. Berg	>1000
*Psidium myrsinites* DC.	292 (212–386)^b^
*Siparuna camporum* (Tul.) A. DC.	251 (207–312)^b^
*Lippia gracilis* Schauer	282 (259–306)^b^

^a^LC_50_ was calculated by probit analysis using SPSS software version 13.0.

^b^Confidence interval of 95%; no dead larvae were observed in the negative control, composed of 0.01% DMSO solution; the positive control, 1 mg/L temefos, exhibited 100% larval mortality.
